# Nurses’ knowledge and therapeutic communication in hospital

**DOI:** 10.1590/0034-7167-2022-0617

**Published:** 2023-11-27

**Authors:** Magdalena Jublina Moykari, Mareta Rodame Sinaga, Vanessa Sakke’, Ineke Patrisia, Chriska Roully Adeline Sinaga

**Affiliations:** I Universitas Pelita Harapan. Tangerang, Banten, Indonesia

**Keywords:** Application, Communication, Knowledge, Nurse, Therapeutic, Aplicação, Comunicação, Conhecimento, Enfermeira, Terapêutica, Aplicación, Comunicación, Conocimiento, Enfermera, Terapéutica

## Abstract

**Objectives:**

to identify the correlation of nurses’ knowledge with the application of nurses’ therapeutic communication in hospitals.

**Methods:**

a descriptive quantitative, correlational design with a total sampling method was used in the study. Respondents were 68 nurses working in an inpatient room in one of the general hospitals in western Indonesia. Modified questionnaires were used in data collection. Analysis of the Pearson chi-square test was used in data analysis.

**Results:**

nurses with sufficient and poor knowledge have a good application of therapeutic communication. There was no correlation between knowledge and the application of nurses’ therapeutic communication.

**Conclusions:**

therapeutic communication is influenced by many factors, but nurses’ knowledge should be maintained and improved to provide holistic care and increase patient satisfaction.

## RESUMO

**Objetivos:**

identificar a correlação do conhecimento dos enfermeiros com a aplicação da comunicação terapêutica por enfermeiros em hospitais.

**Métodos:**

estudo descritivo, quantitativo, correlacional com um método de amostragem total. Os entrevistados foram 68 enfermeiros que trabalhavam na enfermaria em um hospital geral no oeste da Indonésia. Questionários modificados foram usados na coleta de dados. A análise do teste qui-quadrado de Pearson foi utilizada na análise de dados.

**Resultados:**

enfermeiros com conhecimento suficiente e insuficiente têm boa aplicação da comunicação terapêutica. Não foi encontrada correlação entre o conhecimento e a aplicação da comunicação terapêutica pelos enfermeiros.

**Conclusões:**

a comunicação terapêutica é influenciada por muitos fatores, mas o conhecimento dos enfermeiros deve ser mantido e melhorado para prestar cuidados holísticos e aumentar a satisfação do paciente.

## RESUMEN

**Objetivos:**

identificar la correlación entre el conocimiento de los enfermeros y la aplicación de la comunicación terapéutica de los enfermeros en los hospitales.

**Métodos:**

estudio descriptivo, cuantitativo, correlacional con método de muestreo total. Los encuestados fueron 68 enfermeros que trabajaban en una sala del hospital. Se utilizaron cuestionarios modificados para recopilar datos. Los datos se analizaron mediante el análisis de la prueba de chi-cuadrado de Pearson.

**Resultados:**

los enfermeros con conocimientos suficientes y pobres tienen una buena aplicación de la comunicación terapéutica. No hubo correlación entre el conocimiento y la aplicación de la comunicación terapéutica.

**Conclusiones:**

la comunicación terapéutica está influenciada por muchos factores. El conocimiento de los enfermeros debe ser mantenido y mejorado para brindar una atención integral y aumentar la satisfacción del paciente.

## INTRODUCTION

Therapeutic communication is a consciously planned communication process for the patient’s recovery^(1)^. Nurses will encourage patients to express feelings, thoughts, and perceptions associated with visible behavior when communicating. Furthermore, it can foster empathy and caring attitudes, provide service satisfaction, prevent dangerous patient problems, and increase views on nurses and hospitals^(2)^. Inappropriate therapeutic communication impacts nursing actions and makes them less effective, improving the patient’s emotional experience and affecting their recovery rate^(3)^.

According to the Indonesian Ministry of Health^(4)^, the problem development of nurse service satisfaction is increasing in Indonesia. The data result at the East Java Hospitals showed that 83% of patients were dissatisfied with nurse communication services. The study in 2019 by Dora *et al.* ^(5)^ also showed that nurses’ ineffective therapeutic communication causes patient dissatisfaction in the inpatient rooms.

A study on nurses in Indonesia in 2017 showed that 57.1% needed to improve at implementing therapeutic communication^(6)^. Another survey by Mahendro^(7)^ found that 38.14% of nurses with good categories in the orientation phase, 33.57% with good categories in the work phase, and 36.43% with good categories in the termination phase. Research shows that there is no pre-interaction phase by nurses. One of the therapeutic communication application phases is the pre-interaction phase. According to Sarfika *et al.* ^(2)^, nurses will collect and study patient data before visiting patients in the pre-interaction phase. Thus, it is necessary to see whether there are causal factors that influence nurses in the application of therapeutic communication.

Many factors can affect the implementation of therapeutic communication phases. According to Mundakir^(1)^, several factors can influence therapeutic communication, including knowledge, perceptions, values, emotions, background, roles and relationships, and environmental conditions. A Syofyan^(8)^ study revealed that 40.4% of nurses were in a good category in therapeutic communication, and the most influential variable is knowledge. In contrast, the unaffected related variables were environment, emotions, and values. According to research by Handayani *et al.* ^(9)^, variables that show a relationship with nurse therapeutic communication are knowledge, with a *p-value* of 0.045, and an attitude or value variable with a *p-value* of 0.019, where good knowledge and attitudes or values of good nurses will make nurses more skilled in a therapeutic communication application. This result showed that knowledge is the most significant factor in therapeutic communication.

Researchers have observed and conducted a pilot study with five nurses in the inpatient room during clinical practice. The observations found that nurses did not provide patients with information regarding the goals and action steps, did not introduce themselves to the patient, did not keep communicating when doing intervention with the patient, and did not evaluate the patient’s response after taking action. The pilot study found that four nurses had poor knowledge (<56%), three nurses had sufficient knowledge (56-75%), and there were no nurses who had good knowledge related to the four phases of therapeutic communication.

In this sense, it is necessary to identify the nurses’ knowledge that affects therapeutic communication in the hospital, improving nursing care and patient satisfaction. In this regard, questions arise “what level of nurses’ knowledge influences the application of therapeutic communication?”.

## OBJECTIVES

To identify the nurses’ knowledge about therapeutic communication, the application of nurses’ therapeutic communication, and the correlation of nurses’ knowledge with the application of nurses’ therapeutic communication at one of the general hospitals in western Indonesia.

## METHODS

### Ethical aspects

The research obtained ethical clearance from the Research Ethics Committee Faculty of Nursing, Universitas Pelita Harapan, and got permission from the general hospital.

### Design, period, and place of study

The study used the descriptive quantitative correlation method. An equator instrument (STROBE) was used to guide the methodology. The data collection period was between March to April 2022. It took place at one of the general hospitals in western Indonesia.

### Population and sample: inclusion and exclusion criteria

The population consisted of 68 nurses who worked in inpatient rooms, and total sampling was used in this study.

### Study protocol

To carry out this study, two instruments were used. A modified Safitri’s Questionnaire^(10)^ measured the nurses’ knowledge, and Syofyan’s Questionnaire^(8)^ measured the application of nurses’ therapeutic communication. The questionnaire included demographic data, academic level, work experience, therapeutic communication training, the application of therapeutic communication statements, and knowledge questions. Both questionnaires were tested for their validity and reliability. The Cronbach alpha was 0.922 for the application of therapeutic communication and 0.708 for the knowledge, which means that both questionnaires are valid and reliable.

The therapeutic communications questionnaire consisted of 18 statements and provided Likert scale responses (four scales) with choices of never, sometimes, often, and always. The knowledge questionnaire consisted of 14 questions using the Guttman scale. If the answer is correct, given a value of 1, and if the wrong answer is given, it is assigned a value of 0; the following category of the correct answers is 76-100%, which indicates a good level, and the correct answer is 56-75% indicating a sufficient level. The correct answer is less than 56% indicating a poor level.

Permission from the hospital related to the study allowed the authors to distribute the link questionnaires to all nurses in the inpatient rooms through an online platform (WhatsApp). All 68 nurses agreed to join the study and gave informed consent, available online. Afterward, each nurse could access the questionnaire to be filled out and complete all the given questions. The questionnaire was available for completion from March to April 2022.

### Analysis of results and statistics

The study was carried out in univariate and bivariate data analysis. The data were analyzed in the Statistical Package for the Social Sciences (SPSS) using the Kolmogorov Test to test the normality of the data distribution. The data is usually distributed if the *r-alpha* value is > 0.05. The normality test found that the data were normally distributed with an *r-alpha* > 0.05 (0.200 > 0.05).

In descriptive statistics, frequencies and percentages were calculated for both variables, the nurses’ knowledge and the application of therapeutic communication. In bivariate statistics, the correlation between the nurses’ knowledge and the application of therapeutic communication was assessed using the Chi-square statistical test by looking at the Asymptotic Significance of the Likelihood Ratio.

## RESULTS

The characteristics of the respondents are described in Table 1.

**Table 1:** Distribution of respondents’ characteristics (N=68)

Characteristics	Frequency (n)	Percentage (%)
Age (years old) 21-35	68	100
Gender Male Female	11 57	16.2 83.8
Academic level Nursing Diploma Bachelor of Nursing Registered Nurse	13 1 54	19.1 1.5 79.4
Working experience (years) 0-2 3-5 6-10	26 32 10	38.2 47.1 14.7
Therapeutic Communication Training Yes No	44 24	64.7 35.3

The participants of this study were primarily women (83.3%) aged between 21-35 years old. Regarding the academic level, 79.4% of respondents were Registered Nurse (RN). Moreover, most respondents (85.3%) had less than six years of experience working in an inpatient room, and 64.7 of the respondents had joined the therapeutic communication training.

The level of nurses’ knowledge can be seen in Table 2. The study revealed that most nurses in the inpatient room had good knowledge (83.8%), and 13,2% of nurses had sufficient knowledge.

**Table 2:** Distribution of nurses’ knowledge in hospitals (N=68)

Knowledge	Frequency (n)	Percentage (%)
Good	57	83.8
Sufficient	9	13.2
Poor	2	2.9

The application of therapeutic communication can be seen in Table 3. The study revealed that most nurses in the inpatient room were good at applying therapeutic communication (98.5%).

**Table 3:** Distribution of application of therapeutic communication in hospital (N=68)

Application Therapeutic Communication	Frequency (n)	Percentage (%)
Good	67	98.5
Not good	1	1.5

The correlation between nurses’ knowledge and therapeutic communication can be seen in Table 4.

**Table 4:** Correlation of nurses’ knowledge with application of therapeutic communication in hospital (N=68)

Knowledge	Application				Total	*p value*
	Good	%	Not good	%		
Good	56	98.2	1	1.8	57	.837
Sufficient	9	100	0	0	9	
Poor	2	100	0	0	2	

There was no significant association between nurses’ knowledge and the application of therapeutic communication. In Table 4, nurses with sufficient and poor knowledge also applied therapeutic communication well.

## DISCUSSION

Knowledge results from knowing about an object that is known through the senses. This study’s results stated that most nurses had good knowledge about therapeutic communication. Nurses’ good knowledge might result from academic level and therapeutic communication training. Most nurses with good knowledge might have practiced therapeutic communication during the academic process and had refreshment training from the hospital^(11)^. The method of education provides a valuable opportunity for developing someone’s knowledge and abilities. Based on Coelho *et al.* ^(12)^ one process of learning therapeutic communication using an application affects understanding compared to traditional learning. Even though nine nurses had sufficient and two had poor knowledge levels, further assessment is essential as it might affect their performance at work related to patients’ satisfaction.

Therapeutic communication is used to build a trusting relationship between the nurse and the patient, achieve optimal nursing care, and as a patient satisfaction indicator^(3)^. This study’s results showed that 98.5% of nurses had applied high-quality therapeutic communication according to the procedure. Muhiyah^(13)^ revealed in her research that nurses with adequate knowledge also had a good application of therapeutic communication.

According to the analysis results in this study, there is no correlation between nurses’ knowledge and the application of therapeutic communication in the inpatient room. The data support this result that 100% of nurses with sufficient and low knowledge can implement therapeutic communication properly. Even though knowledge is the essential factor influencing how someone acts, it is not related to nursing implementation.

Based on Bloom’s taxonomy, adopting behavior goes through 3 stages: knowledge - attitudes - skills. However, behavior change does not always follow these stages. Some people can perform good skills despite not having adequate knowledge and attitudes. Beliefs, values, perceptions, and culture influence a person’s skills and behavior. Both nurses with high, sufficient, and low knowledge levels can implement therapeutic communication properly^(11,14)^.

The results of the study analysis in this study are in line and show alignment with Yulianti & Purnamawati’s^(11)^ research on Dr. Soeratno Gemolong, in which there is no relationship between nurses’ knowledge and the application of therapeutic communication. But the result of Yulianti & Purnamawati’s research stated that there is a relationship between nurses’ perception and the application of therapeutic communication in inpatient room.

This study’s results differ from the research conducted by Efrianty *et al.* ^(15)^ in the inpatient room of Ernaldi Bahar Hospital Palembang. The study’s outcome stated that there was a relationship between knowledge and the implementation of therapeutic communication with a *p-value* = 0.008 (*p-value* < 0.05). This study is in line with research conducted by Handayani & Armina^(16)^ in the inpatient ward of the Raden Mattaher Jambi General Hospital. The study’s result stated a significant relationship between knowledge and application of therapeutic communication with a *p-value* = 0.045 (*p-value* < 0.05).

A proper understanding of therapeutic communication makes nurses have communication strategies. The communication strategy depends on the purpose of the action or intervention to be carried out on the patient^(1)^. This is to Dermani’s^(17)^ statement that therapeutic communication was the most well-known and applied strategy. In this regard, this study says that 98.2% of nurses apply good therapeutic communication, and 1.8% of nurses do not apply therapeutic communication well. For this reason, it can also be interpreted that from a total of 68 nurses who became respondents, 56 nurses had good knowledge and application of therapeutic communication.

### Study limitations

Apart from the results, this study has several limitations, such as all respondents were only from the inpatient room, and the findings need to be generalizable. It would be more varied if the respondents were taken to another room or unit.

### Contributions to nursing and health

The present study contributes to fostering reflections and giving information on hospitals for patients and nursing practice, especially for improving therapeutic communication application in the inpatient room. When identifying nurses’ knowledge, it was found that they understood how a therapeutic communication process was applied and potentially contributed to framing and developing strategies to maintain the nurses’ knowledge and the application of nurses’ therapeutic communication such as seminars, continuous evaluation, set a goal together rewards, and conditioning.

## CONCLUSIONS

The result of this study was that most of the nurses in the inpatient room had good knowledge related to therapeutic communication and had good therapeutic communication application, even though there is no correlation between nurses’ knowledge and the application of therapeutic communication.

Examining nurses’ knowledge, especially those who provide nursing care with therapeutic communication, is essential. It was mentioned that even nurses with sufficient knowledge who provide patient care might have excellent therapeutic communication applications. Therefore, further study related to other factors might be beneficial. Some theories and studies mention that many factors might impact nurses’ application of therapeutic communication, which might be interesting to be studied in future research.

There needs to be involvement from the training center in preparing a therapeutic communication curriculum. It is essential to create innovative teaching strategies to design a professional with the necessary knowledge for therapeutic communication, aiming to improve patient care and prompt recovery.
